# The Fourier-Laplace Transform—A Conjugate Link Between the Material Brain and the Conscious Mind

**DOI:** 10.3389/fnhum.2021.736761

**Published:** 2021-10-15

**Authors:** Erkki J. Brändas

**Affiliations:** Department of Chemistry, Uppsala University, Uppsala, Sweden

**Keywords:** quantum chemistry, open systems, self-references, long-range correlative information, panexperiential materialism, Fourier-Laplace transform

## Abstract

Recent attempts to establish the quantum boundaries of life is pursued. A pre-existing view of quantum biology is supplemented by the formulation of modern advances in theoretical chemical physics and quantum chemistry. The extension to open system dynamics entails a self-referential amplification supporting the signature of life as well as consciousness via long-range correlative information, ODLCI. The associated negentropic coherence permeates hierarchical and functional organization at multiple levels. In this communication we will derive and review one of the most important mathematical tools, i.e., the combined use of the Fourier- and the Laplace transform. It is shown that an underlying operator algebra facilitates the formulation of the conjugate relationship between energy-time and momentum-space. Implications from augmented general dilation analytic operator families provide novel information-based representations and yield, *inter alia*, a thermo-qubit syntax for communication, which are required to support the quantum Darwinian view of life.

## Introduction

The present work is part of a transdisciplinary approach of the mind-brain problem. The principal equations endorse a new mind-brain doctrine, panexperiential materialism contrasting beliefs in emergent consciousness. Although the former might remind on panexperientialism, going all the way back in time (James, 1879, 1890), there is a consequential difference. Panexperiential materialism has a material foundation exhibiting a conjugate relationship between the material brain, evolving the energy-momentum degrees of freedom of its atomic and molecular constituents, while consistently formulate the conscious mind as a conjugate evolution in space-time under steady state conditions. These aspects have been advanced and compared in some detailed scoping in Poznanski and Brändas (2020). Modern sponsors of panexperientialism, for instance (Georgiev, 2020), advocates an attractive quantum information theoretic approach. Even if one attempts to model quantum transport in proteins via generalized Davydov solitons (Georgiev and Glazebrook, 2020), there are always salient issues, such as the actual relevancy of the adiabatic approximation and the conceived remnant of residual quantum superpositions in the enveloping hot and wet environment of the brain.

There exists a lot of confusion regarding the situation in cognitive sciences. Cognitive psychologists have different ideas regarding consciousness and cognition (Solms, 2018). One reason for this confusion rests on the notion of *the hard problem of consciousness* (Chalmers, 1996). Chalmers presents a case against the materialist dogma, reformulating the crucial distinction between the branch of physical phenomena and the experiential domain as previously expressed by Thomas Nagel, viz. *what is it like for a bat to be a bat* (Nagel, 1974). Although this provides an ambiguous picture of the mind-body enigma it has attained a special standing in the study of consciousness as the latter entails the pinnacle of Darwinian evolution. As pointed out by philosophers, physicists, chemists and biologists alike, there is a fundamental gap between the mental and the material domain (Bitbol, 2008). A traditional way to deal with this conundrum brings forward dual-aspect thinking. For instance the non-Boolean logical framework of modern quantum theory suggests Wolfgang Pauli’s idea of a mind-matter complementarity of one holistic reality (Primas, 2003). The possible reduction of the mind to materialistic physical explanations has been reviewed by Atmanspacher, indicating alternatives to physicalism (Atmanspacher, 2014).

Mind-science phenomena, investigated by new technological advances, has promoted neuroscience as a new scientific subdiscipline devoted to the study of the nervous system. In particular the correlations between phenomenal experiences and neural activity (Crick and Koch, 1990) have increasingly been in focus under the banner of *neural correlates of consciousness*, NCC. Nevertheless the search for mind-brain bridging laws, supported by NCC-oriented approaches, has been criticized by Manzotti and Moderato (2010), since, as they claim, there seems to be no proof that any neural activity is sufficient for consciousness.

Even if interesting proposals have been made to incorporate quantum mechanics (e.g., Hameroff and Penrose, 2014) or the suggested links between consciousness and information (Bohm, 1980), there are some severe challenges to consider, e.g., the explanatory gap and the subject—object dilemma (Bitbol, 2020). For the quantum scientist the main obstacle is the quantum decoherence problem that becomes unavoidable as the thermal noise at 310 K threatens to wash out subtle quantum effects indispensable in the organization of energy and overcoming entropy production in the brain. Decoherence controversies have been intense during the years, starting with the implication that cognitive processes should be classical rather than of quantum origin (Tegmark, 1999).

Irrespective of the subtlety of the various allegations to reject the real problem of decoherence, quantum chemical understanding views the temporal process of leaking phase coherence as a quantum-thermal correlative development. In addition to the interaction between the system and its environment, the activity entails self-organization (Chatzidimitriou-Dreismann and Brändas, 1991), under the steady state situation, *dS* = 0 (Nicolis and Prigogine, 1977), where *S* is the entropy of an open system such as the brain. Elaborating on a thermodynamical quantum picture, with the system immune against decoherence, one is able to derive the transition density matrix


$$\rho_{\text{tr}}=\sum_{k=1}^{n-1}\left|\psi_{k}\right\rangle \,\left\langle \psi_{k+1}\right|=\left|\psi \right\rangle \,\text{J}\,\left\langle \psi \right|$$

in terms of the phase-locked quantum states {ψ_*k*_};*k* = 1,2,*n*, where ρ_tr_ is a steady state solution of the Liouville equation fixed at appropriate temperatures and time scales (Brändas, 2019, 2021). The quantum nature of the formulation proves that the logical possibilities of philosophical zombies should be ruled out by the no-cloning theorem (Wotters and Zurek, 1982). Note that the associated matrix representation in the basis |ψ⟩ results in the nilpotent matrix **J**, defined as


$$\text{J}=\left(\begin{array}{ccc} \begin{array}{cc} 0\; & 1\\ 0\; & 0 \end{array}\; & \cdots \; & \begin{array}{cc} 0\; & 0\\ 0\; & 0 \end{array}\\ \vdots \; & \ddots \; & \vdots \\ \begin{array}{cc} 0\; & 0\\ 0\; & 0 \end{array}\; & \cdots \; & \begin{array}{cc} 0\; & 1\\ 0\; & 0 \end{array} \end{array}\right)$$

with the consequences that the resolvent of the degenerate Hamiltonian that builds the Liouvillian, exhibits a higher order pole of dimension *n* with the result that the related propagator generates a polynomial-delayed evolution exhibiting Poissonian characteristics (Brändas, 2019).

In response to the general problems conveyed above, the research agenda, the concepts and shared values of quantum chemistry (Löwdin, 1992, 1998; Brändas, 2017), suggest an alternative to the original physicalist contention, extending objective interactions and molecular correlations to providing a syntax for subjective semantics for higher order communication without endorsing a dualistic view. The derivation of ρ_tr_, constrained by the dimension *n* to the appropriate temperature and time scales will not be detailed here. Suffice it to say that the steady state provides a hiatus for negentropic gain balancing entropy production, and producing a free energy reorganization from heat to useful work, equal in magnitude to the results of irreversible processes and proportional to *dT*. In addition its Bloch thermalized classical canonical form, represented by the Jordan block **J** in the basis |ψ_*k*_⟩, prompts interesting analytic structures on the so-called unphysical sheet of the complex energy plane. This imparts a generalizations of the Fourier relations between the propagator and its resolvent. The derivation of the corresponding Fourier-Laplace transformations accounting for the higher order poles and associated polynomial amendments will be the main undertaking of this contribution, for more details regarding the original derivations and the meromorphic continuation (see Brändas, 1997, 2019).

## The Fourier-Laplace Transform

The Fourier transform is traditionally a unitary operation that takes data from one domain, i.e., a time-based pattern, to another region, e.g., the spectral ingredient. It is one of the most utilized techniques of all times. It does not only decompose a signal into its constituent frequencies, it finds technical applications in most all of modern advances in technology, such as cell phones, audio-, image-, and video files, digital recording, music composition, etc. The transform and its inverse provide a fundamental tool for solving ordinary- and partial differential equations, including mapping conjugate relationships in general operator algebras of modern quantum theory and their quantum chemical extensions, also involving the Laplace transform. Significant developments in spectral analysis in quantum dynamical systems, with spectral information usually recovered from a short time segment, was initiated by Neuhauser (1990). Further, prolate spheroidal wave functions, PSWFs, are exact eigenfunctions of the Finite Fourier Transformation, providing optimal filters by convoluting band limited functions with PSWFs (Levitina and Brändas, 2008, 2009), which can also be exercised in higher dimensions (Larsson et al., 2004). Other well-known applications exhibit the conjugate relation between space-momentum, for instance analyzing and comparing *d*-wave symmetry of a cuprate condensate wavefunction in ***k***-space and real space (Dunne et al., 2017).

The theory of the Fourier Transform provides an enormous field (see e.g., Reed and Simon, 1978), or the fundamental, historical and practical treatments reviewed by Lützen (1982). Our intention is not to supply another review of the subject, rather we will start at a very simple level to prepare an overall idea that includes generalizations to complex mappings between Cauchy representations of meromorphic functions, conjugate pairs of operators, and finally involving the representation of a certain family of non-normal operators. Let us take the usual model of the transform between the correlation function *g*(*t*) and its transform *f*(ω) (assuming standard existence conditions for the integrals).


$$g\,\left(t\right)=\frac{1}{2\,\pi }\,\int_{-\infty }^{+\infty }f\,\left(\omega \right)\,e^{-i\,\omega \,t}\,d\omega$$

$$f\,\left(\omega \right)=\int_{-\infty }^{+\infty }g\,\left(t\right)\,e^{i\,\omega \,t}\,dt$$

where for simplicity one might associate *t* with time and ω with frequency (ℏ = 1). In its discrete form on a finite interval one finds the connection with the standard Fourier series in harmonic analysis. Note also that the Laplace transform can be obtained by replacing integration intervals and variables accordingly (β > 0).


$$g\,\left(\beta \right)=\int_{0}^{\infty }f\,\left(\omega \right)\,e^{-\beta \,\omega }\,d\omega$$

We will later combine variables and intervals into a suitable Fourier-Laplace transform in order to derive and analyze general relations between propagators and resolvents. Note that a direct inversion of the function *f*(ω) ≡ 1 ensues from the δ-function representation.


$$\delta \,\left(u\right)\,\frac{1}{2\,\pi }\,\int_{-\infty }^{+\infty }e^{-i\,u\,t}\,dt$$

to be discussed in more detail below. Its formal operation appears through


$$\int_{-\infty }^{+\infty }g\,\left(t\right)\,e^{i\,\omega \,t}\,dt=\frac{1}{2\,\pi }\,\int_{-\infty }^{+\infty }f\,\left(u\right)\,du\,\int_{-\infty }^{+\infty }e^{-i\,\left(u-\omega \right)\,t}\,dt=f\,\left(\omega \right)$$

**FIGURE 1:**
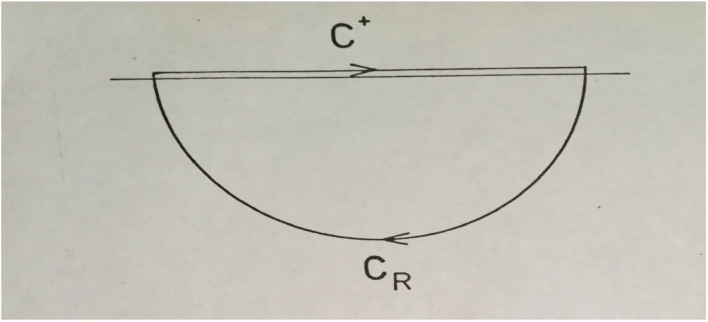
The contour *C*^+^ displayed as the finite interval [−*R*, + *R*] at a finite distance ε above the real axis. By closing the half circle *C_R_*, with radius *R*, in the lower half of the complex plane, the integral can be simply evaluated as the sum of residues of the poles of the actual function inside *C*^+^ + *C*_*R*_.

Let us first consider a simple extension of the frequency ω (or energy) to the field of complex numbers *z* and consider the integral.


$$g\,\left(t\right)=\frac{1}{2\,\pi }\,\int_{C}f\,\left(z\right)\,e^{-i\,z\,t}\,dz$$

where the contour *C* for instance can be chosen (−∞, + ∞) to recover the Fourier relations above. Choosing *C* to run from *0* to + ∞, with *it* = β one recovers the Laplace transform. In order to see how these two transforms will combine we will first study the case, cf. the work of Carleman (1944) discussed and emphasized in Lützen (1982), where below Θ(±*t*) is the Heaviside step function being zero for negative- and one for positive arguments.


(1) $$\mp i\,\Theta \,\left(\pm t\right)=\frac{1}{2\,\pi }\,\int_{C^{\pm }}\frac{e^{-i\,z\,t}}{z}\,dz$$

$$\frac{1}{z}=\int_{-\infty }^{+\infty }\mp i\,\Theta \,\left(\pm t\right)\,e^{i\,z\,t}\,d\,t$$

where the contour *C*^+^, is depicted in Figure 1, and *C*^−^ correspondingly running counter clockwise below the real axis and closed in the upper halfplane. We will prove Eq. (1), assuming appropriate convergence conditions, and univocally by the limit *R*→∞.

The second relation of Eq. (1), follows at once for, e.g., *C*^+^, with *z* = ω + *i*ε,ε > 0,


$$\int_{-\infty }^{+\infty }-i\,\Theta \,\left(t\right)\,e^{i\,z\,t}\,d\,t=-i\,{\left[\frac{1}{i\,z}\,e^{i\,\omega \,t}\,e^{-\varepsilon \,t}\right]}_{0}^{\infty }=\frac{1}{z}$$

yielding a result independent of ε > 0, which can easily be combined to


$$\frac{1}{z}=\int_{-\infty }^{+\infty }\mp i\,\Theta \,\left(\pm t\right)\,e^{i\,z\,t}\,d\,t;\left\{ \begin{array}{c} \mathrm{upper}\,\mathrm{sign}⟹\text{im}\,z>0\\ \mathrm{lower}\,\mathrm{sign}⟹\text{im}\,z<0 \end{array}\right.$$

Next, we prove the remaining relation of Eq. (1), by considering the integral with the contour as displayed in Figure 1, with *t*,δ > 0, using the residue theorem (Ahlfors, 1979),


$$\frac{i}{2\,\pi }\,\int_{C^{+}+C_{R}}\frac{e^{-i\,\left(z+i\,\delta \right)\,t}}{z+i\,\delta }\,dz=-2\,i\,\pi \,\frac{i}{2\,\pi }=1$$

One realizes that closing the contour in the lower complex halfplane leads to


$$\lim_{\begin{array}{c} \left|z\right|\to \infty \\ t>0 \end{array}}\frac{e^{-i\,\left(z+i\,\delta \right)\,t}}{z+i\,\delta }=0$$

finding, with *C* = *C*^+^ + *C*_*R*_ and letting *R*→∞, that


$$\lim_{R\to }\int_{-R}^{R}\frac{e^{-i\,\left(E+i\,\delta \right)\,t}}{E+i\,\delta }\,dE=\underset{R\to }{\text{lim}}\oint_{C}\frac{e^{-i\,\left(z+i\,\delta \right)\,t}}{z+i\,\delta }\,dz=-2\,\pi \,i$$

In ascertaining the last relation we have utilized the following estimates, for arbitrary small θ_0_ > 0


$$\int_{C_{R}}\frac{e^{-i\,\left(z+i\,\delta \right)\,t}}{z+i\,\delta }\,dz=e^{\delta \,t}\,\int_{-\theta_{0}}^{-\pi +\theta_{0}}\frac{e^{-i\,R\,t\,\cos\vartheta }\,e^{R\,t\,\sin\vartheta }}{1+i\,\delta \,R^{-1}\,e^{-i\,\vartheta }}\,i\,d\vartheta +\int_{0}^{-\theta_{0}}+\int_{-\pi +\theta_{0}}^{-\pi }$$

applying the standard change of variables and omitting the details in the next two terms


$$\left\{\begin{array}{c} z=R\,e^{i\,\theta }\\ d\,z=i\,z\,d\,\theta \end{array}\right\}$$

When *R*→∞, the first term vanishes since *sin*⁡θ is negative in [−π < θ < 0]. One obtains for ϑ≤θ_0_, i.e., *sin*⁡θ≈θ that the second term vanishes


$$|\int_{0}^{-\theta_{0}}|\left(<1-\delta R^{-1}\right)\int e^{R\,t\,\theta }d\theta <{\left[\frac{1}{R\,t}e^{R\,t\,\theta }\right]}_{0}^{-\theta_{0}}=\frac{1}{R\,t}\,\left(e^{-R\,t\,\theta_{0}}-1\right)\overset{R\to \infty }{⟶}0$$

and similarly for the third term. In summary for *t* > 0, and writing *z* = *E* + *i*ε, explicitly defining the contour


$$\underset{R\to \infty }{C^{+}=\text{lim}}\left(-R+i\,\delta ,+R+i\,\delta \right)$$

one can express the result as


$$-i\,\left(\Theta \,t\right)=\frac{1}{2\,\pi }\,\int_{C^{+}}\frac{e^{-i\,z\,t}}{z}\,dz$$

Performing a similar analysis for *C*^−^ and *z* = *E*−*i*δ the proof of Eq. (1) is hence completed.

Taking the time derivative one formally gets the Fourier relations between the unit- and the delta function, which under appropriate circumstances represents distributions, working on functions being properly bounded on respective complex halfplanes.


(2) $$\delta \,\left(t\right)=\frac{1}{2\,\pi }\,\int_{C}e^{-i\,z\,t}\,dz$$

$$1=\int_{\infty }^{+\infty }\delta \,\left(t\right)\,e^{i\,z\,t}\,dt$$

**FIGURE 2:**
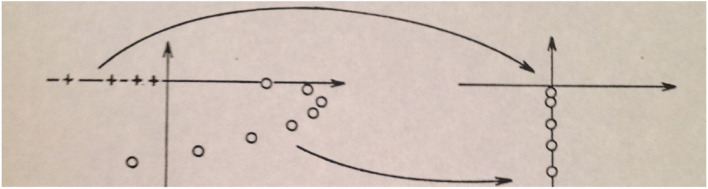
The left part of the figure shows an archetypical spectrum of an operator, *H*, with the point spectrum σ_*P*_(*H*) along the negative real axis, denoted by *“+,”* and a simple continuous spectrum σ_*AC*_(*H*) along the positive real axis. For later use we have also indicated complex resonance eigenvalues in the lower complex plane as they appear in realistic physical cases. To the right we have inserted the spectrum of the corresponding Liouville equation (see Obcemea and Brändas, 1983). Here all the bound states pile together at the origin, while the associated resonances fall down on the negative imaginary axis.

In Eq. (2) the contour *C* means *C*^+^ for *t* > 0 and *C*^−^ for *t* < 0. Replacing *z*→(*z*−*H*) gives trivially the formal relations (3) below, if *H* is a real constant. The question is what happens when *H* becomes an operator with a spectral representation as indicated in Figure 2 This begs a clarification, since so-called complex resonance eigenvalues have been added to the spectral classification (Balslev and Combes, 1971). This will be addressed later, but first we will treat the case of a self-adjoint operator *H* with a real spectrum.

From the simple cases discussed above, the extension to represent operators and their associated Fourier relations follow unambiguously. Consider first the retarded-advanced propagator $G_{P}^{\pm }\,\left(t\right)$ and its resolvent *G*_*R*_(*z*)


(3) $$G_{P}^{\pm }\,\left(t\right)=\mp \text{i}\,\Theta \,\left(\pm t\right)\,e^{-i\,H\,t};G_{R}\,\left(z\right)={\left(z-H\right)}^{-1}\;$$

where the operator *H* is a self-adjoint operator, for simplicity, assuming a simple spectrum, σ, consisting of a point spectrum σ_*P*_(*H*) and an absolutely continuous part σ_*AC*_(*H*) (see Figure 2). In the following the operator will represent the Hamiltonian describing a chemical system, such as an atom or a molecule, including Coulomb interactions, the latter without the complications such as the appearance of a singularly continuous spectrum. It is mentioned here because the Coulomb Hamiltonian satisfies the conditions of dilation analyticity (Balslev and Combes, 1971, see also Simon, 1973), comprising the various general situations encountered in quantum chemistry and chemical physics. The rationale of this observation will be clear further below.

With the operator *H* exhibiting the spectral expansion, for an extension to the Liouville picture (see Obcemea and Brändas, 1983),


$$H=\int_{\sigma }E\,d\mu \,\left(E\right)$$

with the spectral measure μ(*E*) simply defined from resolution of the identity *I*, i.e.,


$$I=\int_{\sigma }d\mu \,\left(\omega \right)=\sum_{\sigma_{P}}\left|\psi_{k}\right\rangle \,\left\langle \psi_{k}|+\int_{\sigma_{A\,C}}|\psi \,\left(\omega \right)\right\rangle \,d\,\omega \,\left\langle \psi \,\left(\omega \right)\right|$$

in terms of the eigenvalues of the Hamiltonian, i.e., {ψ_*k*_,ψ(ω)} with ⟨ψ_*k*_|ψ_*l*_⟩ = δ_*kl*_, ⟨ψ_*k*_|ψ(ω)⟩ = 0, and ⟨ψ(ω)|ψ(ω′)⟩ = δ(ω−ω′) spanning the actual Hilbert space. The spectral measure is here accompanied by a spectral function displaying steps at the discrete points in σ_*P*_(*H*) and exhibiting a locally integrable function in the continuum, σ_*AC*_(*H*).

Before navigating the complex plane, one needs to know what happens at the boundary where the limits from each halfplane meet. Introducing the explicit operator representations one gets


(4) $$G_{P}^{\pm }\,\left(t\right)=\mp \text{i}\,\Theta \,\left(\pm t\right)\,\int_{\sigma }e^{-i\,\omega \,t}\,d\mu \,\left(\omega \right);G_{R}\,\left(z\right)=\int_{\sigma }\frac{d\,\mu \,\left(\omega \right)}{z-\omega }$$

finding directly the transforms


(5) $$G_{P}^{\pm }\,\left(t\right)=\frac{1}{2\,\pi }\,\int_{C}G_{R}\,\left(z\right)\,e^{-i\,z\,t}\,dz$$

$$G_{R}=\left(z\right)\,\int_{-\infty }^{+\infty }G_{P}^{\pm }\,\left(t\right)\,e^{i\,z\,t}\,dt;\left\{ \begin{array}{c} \text{t}>0⟺\text{im}\,z>0\\ \text{t}<0⟺\text{im}\,z<0 \end{array}\right.$$

Anticipating complex resonance eigenvalues (Balslev and Combes, 1971, see Figure 2), one realizes that the spectral contour must also be extended to the complex *z*-plane, while observing that analyticity requirements of the Greens function, *G*_*R*_(*z*), being regular in the upper complex half plane, sets up mathematical requirements for analytic continuations into the lower half plane and vice versa for the other half. We will return to these conditions and its consequences below.

In order to turn the abstract operator representations above into a more concrete functional relation we introduce a suitable normalized reference function, φ, in the Hilbert space introduced above, which might be represented as


(6) $$\varphi =I\,\varphi =\sum_{k}c_{k}\,\psi_{k}+\int_{\sigma_{A\,C}}c\,\left(\omega \right)\,\psi \,\left(\omega \right)\,d\omega$$

$$c_{k}=\left\langle \psi_{k}|\varphi \right\rangle \neq 0;c\,\left(\omega \right)=\left\langle \psi \,\left(\omega \right)|\varphi \right\rangle \neq 0$$

From ⟨φ|φ⟩1 follows


$$1=\sum_{k}{\left|c_{k}\right|}^{2}+\int_{\sigma_{A\,C}}{\left|c\,\left(\omega \right)\right|}^{2}\,d\omega =\int_{\sigma }d\rho \,\left(\omega \right)$$

where we have introduced the Stieltjes integral via the spectral function ρ with jumps |*c*_*k*_|^2^ at the points ω_*k*_ of σ_*P*_ and represented by the continuous function |*c*(ω)|^2^ at σ_*AC*_.

The operator relations above can now be represented as


$$g^{\pm }\,\left(t\right)=\left\langle \varphi |G_{P}^{\pm }\,\left(t\right)|\varphi \right\rangle =\mp \text{i}\,\Theta \,\left(\pm t\right)\,\int_{-\infty }^{+\infty }e^{-i\,\omega \,t}\,d\rho \,\left(\omega \right)$$

$$f\,\left(z\right)=\left\langle \varphi |G_{R}\,\left(z\right)|\varphi \right\rangle =\int_{-\infty }^{+\infty }\frac{d\,\rho \,\left(\omega \right)}{z-\omega }$$

In order to study the spectral function in more detail, we will consider the integral below at a point *E* in σ_*AC*_


(7) $$f\,\left(E+i\,\varepsilon \right)=\sum_{k}\frac{{\left|c_{k}\right|}^{2}}{E+i\,\varepsilon -E_{k}}+\int_{-\infty }^{+\infty }\frac{{\left|c\,\left(\omega \right)\right|}^{2}\,\left(E-\omega \right)\,d\,\omega }{{\left(E-\omega \right)}^{2}+\varepsilon^{2}}-i\,\int_{-\infty }^{+\infty }\frac{{\left|c\,\left(\omega \right)\right|}^{2}\,\varepsilon \,d\,\omega }{{\left(E-\omega \right)}^{2}+\varepsilon^{2}}$$

Taking the limit ε→0 + 0 one obtains


(8) $$\lim_{\varepsilon \to 0+0}\int_{-\infty }^{+\infty }\frac{d\,\rho \,\left(\omega \right)}{E+i\,\varepsilon -\omega }=𝒫\,\int_{-\infty }^{+\infty }\frac{{\left|c\,\left(\omega \right)\right|}^{2}\,d\,\omega }{E-\omega }-i\,\pi \,{\left|c\,\left(E\right)\right|}^{2}$$

where 𝒫 denotes the Cauchy principal value of the integral, i.e., from the second and third terms of Eq. (7). The expression, Eq. (8), signifies a so-called Kramers-Kronig relation employed to relate the real and imaginary parts of a complex function, such as a physical response function or an electric susceptibility, etc. Note that the interesting information comes from the evaluation of the function |*c*(ω)|^2^ as it is defined in σ_*AC*_, while the jumps are determined by |*c*_*k*_|^2^ above.

A simple proof of the relation involving the imaginary part of (8) follows from the simple fact that


$$\frac{\varepsilon }{x^{2}+\varepsilon^{2}}\overset{\varepsilon \to +0}{⟶}\pi \,\delta \,\left(0\right)$$

which can be derived as follows. Consider a general function *f*(*x*), which decays appropriately in the complex plane (see Figure 1), and stays finite on the real axis. If *C* is a contour running from −*R* to + *R* and closed in the upper half plane with an analogous result obtained if the contour runs in the lower half plane, one finds


$$\int_{-\infty }^{+\infty }\frac{\varepsilon \,f\,\left(x\right)\,d\,x}{x^{2}+\varepsilon^{2}}=\underset{R\to }{\text{lim}}\int_{C}\frac{\varepsilon \,f\,\left(z\right)\,d\,z}{\left(z+i\,\varepsilon \right)\,\left(z-i\,\varepsilon \right)}=\pi \,f\,\left(i\,\varepsilon \right)\overset{\varepsilon \to +0}{⟶}\pi \,f\,\left(0\right)$$

The result involves the following limiting procedure of the integral over *C_R_*


$$\int^{\text{​}}\frac{\text{ε}f(z)dz}{z^{2}+{\text{ε}}^{2}}=\left\{\begin{array}{c} z=Re^{i\theta }\\ dz=zd\theta \end{array}\right\}=\int_{0}^{\text{π}}\frac{\text{ε}f(Re^{i\theta })d\text{θ}}{\left(Re^{i\theta }+\frac{{\text{ε}}^{2}}{Re^{i\theta }}\right)}\vec{R\to }\;\infty^{0}$$

The other limit ε→0−0 follows, as said, trivially. Replacing *x*→(*E*−ω), Eq. (8) follows. Symbolically one can now write the general operator equations


$$G_{R}\,\left(E\pm i\,0\right)=𝒫\,\int_{\sigma }d\mu \,\left(\omega \right)\,{\left(E-\omega \right)}^{-1}\mp i\,\pi \,\delta \,\mu \,\left(E\right)$$

or


$$G_{R}\,\left(E\pm i\,0\right)=𝒫\,{\left(E-H\right)}^{-1}\mp i\,\pi \,\delta \,\left(E-H\right)$$

corresponding to the retarded-advanced propagator defined in Eq. (5)


$$G_{P}^{\pm }\,\left(t\right)=\mp \text{i}\,\Theta \,\left(\pm t\right)\,\int_{\sigma }d\mu \,\left(\omega \right)\,{\text{e}}^{-i\,\omega \,t}$$

These operator representations are related through the Fourier transforms Eq. (4). The step from functions to operators have been reduced to a technicality in terms of an appropriately defined spectral function. Since we have separated the retarded and advanced parts one is able to transverse the complex plane under straightforward assumptions of asymptotically decaying functions in the appropriate complex halfplanes. Finally we note that the so-called causal Greens function can be written $G=+i\,G_{P}^{+}\,\left(t\right)-i\,G_{P}^{-}\,\left(t\right)=e^{-i\,H\,t}$. Summarizing we have derived the following Fourier transforms between the propagator *G*_*P*_(*t*) and the resolvent *G*_*R*_(*z*)


(9) $$G_{P}^{\pm }\,\left(t\right)=\frac{1}{2\,\pi }\,\int_{C}G_{R}\,\left(z\right)\,e^{-i\,z\,t}\,dz$$

$$G_{R}\,\left(z\right)=\int_{-\infty }^{+\infty }G_{P}^{\pm }\,\left(t\right)\,e^{i\,z\,t}\,dt$$

where the retarded-advanced form of (9) guarantees that the analyticity requirements, i.e., referring to the appropriate complex halfplane matching the proper time direction. As we will make use of the complex plane, with functional properties relating to each halfplane separately, we will refer to the Fourier-Laplace transform in what follows.

Let us summarize the formulas above and rewrite the Schrödinger equation in a slightly more general form, i.e., first as a time dependent equation, which in its retarded-advanced form contains an inhomogeneous memory term ψ(0)


$$\begin{array}{c} \left(i\,\frac{\partial }{\partial t}-H\right)\,\psi^{\pm }\,\left(t\right)=\pm \delta \,\left(t\right)\,\psi \,\left(0\right)\\ \psi^{\pm }\,\left(t\right)=\pm i\,G^{\pm }\,\left(t\right)\,\psi \,\left(0\right) \end{array}$$

and the time independent Fourier related equation


$$\begin{array}{c} \left(z-H\right)\,\psi^{\pm }\,\left(z\right)=\pm i\,\psi \,\left(0\right)\\ \psi^{\pm }\,\left(z\right)=\pm i\,G\,\left(z\right)\,\psi \,\left(0\right) \end{array}$$

Note that in principle there could be different limits as ψ^±^(*t*)*t*→0. A general operator form reads


$$\left(i\,\frac{\partial }{\partial t}-H\right)\,G^{\pm }\,\left(t\right)=\delta \,\left(t\right)$$

and


(z-H)⁢G⁢(z)=I

which for *E* ∈ σ_*AC*_(*H*) becomes subjected to the principal value form, Eq. (8).

In the past several efforts to extend quantum theory, i.e., the Schrödinger equation, to the non-self-adjoint case have been made. A mathematically rigorous, and suitable extension was given by the Balslev-Combes theorem for dilation analytic operators (Balslev and Combes, 1971, see also Simon, 1973; Reed and Simon (1978). The theory fortunately includes the long range Coulomb potential of molecular physics and quantum chemistry. Advanced applications and texts attest to this circumstance (Nicolaides, 2010; Moiseyev, 2011).

**FIGURE 3:**
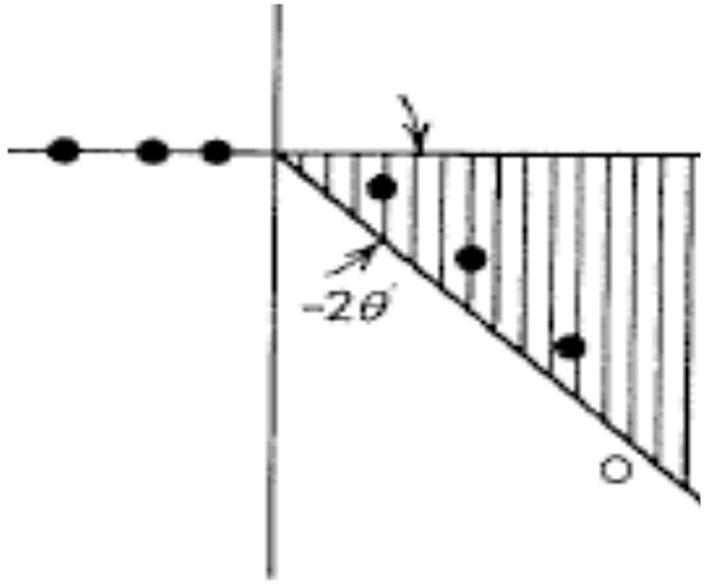
A portrayal of the simple case of the Hamiltonian, *H*, exhibiting bound states, black dots on the negative real energy axis, with continuous spectrum, σ_*AC*_(*H*), rotated −2θ around the origin, displaying a sector, indicated with stripes and arrows, where the resolvent *G*_*R*_(*z*), Eq. (4) has a meromorphic continuation.

**FIGURE 4:**
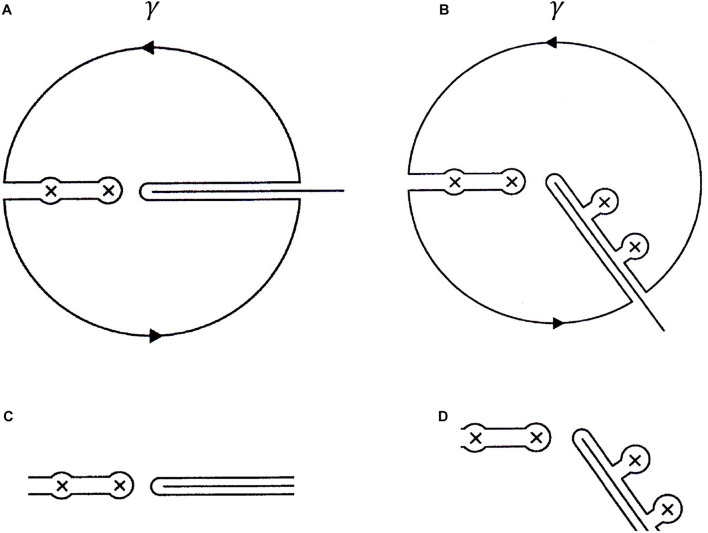
The contour γ defining the Cauchy representation of *f*(*z*) in the case **(A)** referring to the situation displayed in Eqs. (7, 8) and in the case **(B)** after analytic continuation. The excluded regions, **(C,D)** are adapted after the spectral domains in each case.


In essence, for details see e.g., Reed and Simon (1978), the generalization has its root in an extension of the unitary scaling operator, *U*(η), to comprise complex dilation parameters η = |η|*e*^*i*θ^, 0≤θ < θ_0_ for some θ_0_, depending on the actual potential. This operator brings about a rotation −2θ of the real line corresponding to σ_*AC*_(*H*), i.e., the positive real axis in Figure 2. The result is depicted in Figure 3. Note that the discontinuity in *G*_*R*_(*z*), as *z* crosses the positive real axis, from above or below, as indicated by the continuous cut along the positive real axis, has been transferred to a line rotated −2θ around origo. The dots in the striped area represents complex resonance eigenvalues of the complex scaled Hamiltonian *H*(η) = *U*(η)*HU*(η)^−1^, displaying one uncovered resonance indicated by the small circle. In this situation the transformed spectral expansion is represented by the bound states, as before, plus the sum of the exposed resonances states and finally the contributions from the integration over the rotated cut. For more details and numerical results, employing Weyl’s theory for singular second-order differential equations, originally adapted to quantum chemistry and reviewed by Hehenberger et al. (1974), we refer to Engdahl et al. (1986; 1988). The new situation calls for a reorganization of the integration contour displayed below in Figures 4A,C to be altered as indicated in Figures 4B,D. Since *f*(*z*) is assumed to decay as |*z*|→∞, one can let the path γ extend to infinity and cover the whole complex plane except where the function exhibits singular behavior. The contour can also approach the real line arbitrarily close without touching σ. In Figure 4 one also observes that the paths joining the bound state eigenvalues and the ones encircling the complex resonances to and from the rotated cut will not contribute to the path integral [we have briefly employed the path −γ to get the correct sign in Eq. (10)]

(10) $$f\,\left(z\right)=\left\langle \varphi |G_{R}\,\left(z\right)|\varphi \right\rangle =\int_{\sigma }\frac{d\,\rho \,\left(\omega \right)}{z-\omega }$$

$$\frac{1}{2\,\pi \,i}\,\oint_{-\gamma }f\,\left(z\right)\,dz=\int_{\sigma }d\rho \,\left(\omega \right)$$

Note that the resonance eigenvalue may be complex, i.e., *z*_*i*_ = *E*_*i*_−*i*ε_*i*_ with the physical interpretation of the imaginary part ε_*i*_ = ℏ/2τ usually being inversely related to the lifetime τ of the state.

In order to establish the generalized picture anticipated in Figures 2, 3, we return to the function *f*(*z*) defined and represented in Eqs. (7, 8). Using Cauchy’s integral formula, with γ defined in Figures 4A,B below, *f*(*z*) and its derivatives, can be represented as, (with *z* inside γ)


$$f\,\left(z\right)=\frac{1}{2\,\pi \,i}\,\oint_{\gamma }\frac{f\,\left(\zeta \right)}{\zeta -z}\,d\zeta ;f^{n}\,\left(z\right)=\frac{n!}{2\,\pi \,i}\,\oint_{\gamma }\frac{f\,\left(\zeta \right)}{{\left(\zeta -z\right)}^{n+1}}\,d\zeta$$

Using the residue theorem and the Cauchy principal value formula for *z* approaching σ_*AC*_, one obtains the contributions to *f*(*z*) corresponding to the contours Figures 4C,D. Note that Eq. (10) can easily by continued to the cases **B**) and **D**). It is moreover interesting that the case **D**) displays additional resonance eigenvalues “pulled from the continuum” with the result that the generalized spectral density gets deflated at the interval correspondingly below the resonances, while it becomes that of a free particle asymptotically, for details including the behavior of typical pole strings (see Engdahl et al., 1986, 1988). Another interesting feature is the appearance of multiple eigenvalues (Natiello et al., 1987, see also Figure 2.11 in Brändas, 2012).

In fact the relations derived above adapt, as pointed out, with minor changes to the situation where the contours *C* have been adjusted accordingly (Brändas, 1997). However, as already mentioned, there is the difficulty of multiple eigenvalues and their block structure that applies to general non-normal operators, i.e., those that do not commute with their own adjoints. This seriously complicates the matrix problem, since it introduces degeneracies associated with irreducible matrix blocks, which in its classical canonical form is represented by the unit matrix times the degenerate eigenvalue superimposed on *n*-dimensional matrix blocks, **J**, with zeroes along the main diagonal and with the super-diagonal composed of ones. As we will see below, this appears to be a blessing in disguise, creating novel possibilities to map complex enough systems in biology at far from equilibrium situations.

In principle we have obtained a general Fourier-Laplace relation between the propagator $G_{P}^{\pm }\,\left(t\right)$ and the resolvent *G*_*R*_(*z*) as given by Eq. (9). The degenerate situation is simply incorporated in the standard self-adjoint picture, since the degeneracy of an eigenvalue is trivially characterized by diagonal operators, i.e., with the degenerate eigenvalue multiplied by the unit operator *I*, represented as a unit matrix in the space spanned by the degenerate eigenfunctions of *H*. Hence for *H* = *EI* one obtains directly the formal relationship


(11) $$\frac{1}{2\,\pi \,i}\,\oint \frac{e^{-i\,z\,t}}{z\,I-H}\,dz=\text{Res}\,\left(H\right)=e^{-i\,H\,t}=e^{-i\,E\,t}\,I$$

where we have, without restricting the problem, chosen the contour of the line integral to be the unit circle in the complex plane with the origin at *E* and running counter clockwise. Since we are dealing with dilation analytic operators, *E* could here be complex with a negative imaginary part.

It is straightforward to translate the result to matrix algebra and carry out the steps with results analogous to Eqs. (7–9). Actually, the procedure can in principle be applied to *H*(η), provided resonance poles, uncovered by complex scaling, are simple poles. This begs the question what happens in the general case, where multiple degeneracies appear in the resolvent accompanied by irreducible Jordan blocks of corresponding dimensions? It is customary to characterize the order of the Jordan blocks of a particular degeneracy by its dimensions, i.e., their Segrè characteristics.

Consider the operator *J*, with its *n*-dimensional matrix representation *J*, as defined above. As a result the corresponding operator *J* is nilpotent, i.e.,


$$J^{n-1}\neq 0,J^{n}=0$$

with the order, *n*, yielding the Segrè characteristic associated with *J*, or the corresponding matrix *J*. Adding a nilpotent part to *H*, i.e.,


H⁢(η)=E⁢I+α⁢J

where α is a complex number given by the nature of the physical problem, the aforementioned formal procedure Eq. (11) would, after expansion of the exponential and the inverse around *z* = *E* give


(12) $$e^{-i\,H\,\left(\eta \right)\,t}=\frac{1}{2\,\pi \,i}\,\oint \frac{e^{-i\,z\,t}}{z\,I-H\,\left(\eta \right)}\,dz=e^{-i\,E\,t}\,\left(I-i\,\alpha \,J\,t\right)=e^{-i\,E\,t}\,e^{-i\,\alpha \,J\,t}$$

where for simplicity we have taken the Segrè characteristic to be *n* = *2*. Note that one should have obtained the same result by mnemonically determine the residue, Res(γ,*H*), by formally inserting *H*(η) even if it is a non-normal operator containing a nilpotent part *J*. This is clearly consistent with the original definition and separate evaluation of the resolvent and the propagator, which both trivially exhibits finite operator expansions due to the nilpotent property of *J*.

It is interesting to construe Eq. (12) in more detail to realize the consequences of the present operator formulation. For instance introducing two orthonormal degenerate solutions χ_1_,χ_2_, corresponding to the degenerate subspace related to the eigenvalue *E* of *H*, one obtains (*n* = 2)


$$I=\left|\chi_{1}\right\rangle \,\left\langle \chi_{1}\right|+\left|\chi_{2}\right\rangle \,\left\langle \chi_{2}\right|;J=\left|\chi_{1}\right\rangle \,\left\langle \chi_{2}\right|$$

Returning to the meromorphic function *f*(*z*) defined in Eqs. (7, 9), where the resolvent has the degenerate structure indicated above, one finds that


$$f\,\left(z\right)=\left\langle \varphi |G_{R}\,\left(z\right)|\varphi \right\rangle ={\left(z-E\right)}^{-1}+\alpha \,\left\langle \varphi |\chi_{1}\right\rangle \,{\left(z-E\right)}^{-2}\,\left\langle \chi_{2}|\varphi \right\rangle$$

exhibits a higher order pole. Employing again the Fourier-Laplace transformation


$$g^{\pm }\,\left(t\right)=\frac{1}{2\,\pi }\,\int_{C^{\pm }}f\,\left(z\right)\,e^{-i\,z\,t}\,dz$$

one obtains


(14) $$g^{\pm }\,\left(t\right)=\mp \text{i}\,\Theta \,\left(\pm t\right)\,e^{-i\,E\,t}\,\left\{1-i\,\alpha \,t\,\left\langle \varphi |\chi_{1}\right\rangle \,\left\langle \chi_{2}|\varphi \right\rangle \right\},$$

commensurate with Eq. (12).

One general way to identify hidden degeneracies would be to employ the argument principle. In the case above one finds


$$\frac{1}{2\,\pi \,i}\,\oint_{\gamma }\frac{f^{\prime }\,\left(z\right)}{f\,\left(z\right)}\,dz=-2$$

a winding number of –2, matching the Segrè characteristic, *n* = 2, of the degenerate eigenvalue *E*. Hence, one infers that the inverse Fourier transform *g*(*t*) contains an extra term that suggests a more fundamental origin, i.e., an operator contribution from −*i*α*Jt* in agreement with Eq. (12). Although a lot has been achieved in terms of hyperfunctions and the field of algebraic geometry since 1981, it is interesting to learn of the skepticism offered by pioneers like Courant-Hilbert that *in the realm of ideal functions not all operations of classical calculus can be carried out* (Lützen, 1982). It is also quite thought-provoking to observe that the present formulation displays a close connection between the theory of distributions, according to Carleman (1944), and generalized spectral theory (Balslev and Combes, 1971).

In what follows we will review some of the mathematical problems that arise when formalized operator algebra is detailed with a more rigorous mathematical grounding. The aim, among other things, is to find a provisional license for building consistent models, extending the formulation to include biological systems and their Darwinian evolution. An interesting analogy, which will not be detailed here, is the mapping of Gödel’s celebrated link between model- and proof theory (Gödel, 1931), providing a logical table that translates a self-referential proposition into a Jordan block of linear algebra (Brändas, 2015). The correspondence is carried further as it reverberates with a simple operator algebra treatment, commensurate with Einstein’s equivalence principle. This surprising correlation reflects an intrinsic self-referential characteristic of a living system authorizing degenerate maps, i.e., Jordan blocks of a specific order, *n*, depending on time-temperature constraints, as self-organizing units of life forms and evolving organisms and their communication. This gives an alternative interpretation of Gödel’s celebrated result that formal axiomatic systems are inherently limited. Finally we will present some basic applications that exhibits the relationship between general operator relations, Eq. (9), formally interpreted as Fourier-Laplace duals, suggesting isomorphic connections between material systems and their abstract spatial and temporal evolutions.

## Localization of Complex Resonances and Higher Order Poles

In the previous section, we have derived general operator relations and their functional relations incorporating general analytic structures, i.e., essentially of analytic functions in the upper complex halfplane with a non-negative imaginary part to be meromorphically continued to the lower halfplane (Nevanlinna, 1953). In this section we will demonstrate how to localize and verify the associate pole strings and their properties. We have already referred to Balslev-Combes’ theorem for dilation analytic operators (Balslev and Combes, 1971; Reed and Simon, 1978), in order to obtain a rigorous extension of quantum dynamics to incorporate a non-self-adjoint formulation, with dilated operators, complex eigenvalues, and other consequences, such as operator domains and ranges, dense and closed subsets, dilated conjugate relations, etc. The need for these extensions is quite obvious since otherwise there might appear some unexpected results that may sound contradictive. One concerns the Feshbach-Fano partitioning in scattering theory, second the consequences of the domain restrictions of the scaling operator and the contractive semigroup properties of the generator of time evolution, and third the manifestation of Jordan blocks and their significance.

The celebrated Feshbach-Fano technique to find scattering solutions of the Schrödinger equation, albeit very powerful, is not exact as to the localization of the actual position of the resonance. Selecting a dilatation analytic Hamiltonian, *H*, a reference function φ, not an eigenfunction, nor orthogonal to the spectrum, of *H*, with the projection operators *O* = |φ⟩⟨φ|,*P* = *I*−*O*, and the reduced resolvent *T*(*z*) = *P*(*z*−*PHP*)^−1^*P*, a straightforward operator algebra yields rigorous tools to unify the treatment of both bound and quasi-bound states; for more details see Micha and Brändas (1971). To elaborate on the reasoning we rewrite the Schrödinger equation in the Löwdin wave-operator formalism (Löwdin, 1968)


(z-H)⁢Ψ⁢(z)=(z-h⁢(z))⁢φ

with the trial wave function Ψ(*z*) = (*I* + *T*(*z*)*H*)φ and the bracketing function given by *h*(*z*) = ⟨φ|*H* + *HT*(*z*)*H*|φ⟩, noticing that an eigensolution to the differential equation for *z* = *E*_*b*_ ∈ σ_*P*_ is obtained from


$$z=h\,\left(z\right)=E_{b}$$

$$\Psi \,\left(E_{b}\right)=\left(I+T\,\left(E_{b}\right)\,H\right)\,\varphi$$

$$H\,\Psi =E_{b}\,\Psi$$

The name “bracketing function” refers to the bracketing property of *h*(*z*), i.e., inserting an upper bound to *E_b_* in the function yields a lower bound and vice versa. Note that Ψ(*z*) is subject to intermediate normalization and therefore not normalized,


⟨Ψ⁢(z)|φ⟩=1;⟨Ψ⁢(z)|Ψ⁢(z)⟩=1+⟨T⁢(z)⁢H⁢φ|T⁢(z)⁢H⁢φ⟩

It is interesting to document what happens when *z*→*E* ∈ σ_*AC*_(*H*), celebrating the principal value relation discussed in Eq. (8), applied to the reduced resolvent *T*(*z*)


$$\lim_{z\to E+i\,0}P\,{\left(z-P\,H\,P\right)}^{-1}\,P=𝒫\,{\left(E-P\,H\,P\right)}^{-1}\,P-i\,\pi \,\delta \,\left(E-P\,H\,P\right)$$

yielding


$$h\left(E+i0\right)=\left\langle \varphi |H+H𝒫{\left(E-PHP\right)}^{-1}PH|\varphi \right\rangle -i\pi \left\langle \varphi |H\delta \left(E-PHP\right)H|\varphi \right\rangle$$

demonstrating that *h*(*E*) is now a complex function with a negative imaginary part.

Summarizing we have *h*^+^(*E*) = *E*−*i*ε = *E*−*i*Γ/2, where Γ is the (Fermi Golden Rule) level width reciprocally related to the life time τ. To find a complex resonance eigenvalue, fulfilling *h*(ε_*s*_) = ε_*s*_ = *E*_*s*_−*i*Γ_*s*_/2 one needs to solve the equations (Micha and Brändas, 1971)


(13) Es=Re⁢h+⁢(Es-i⁢Γs2)Γs=-2⁢Im⁢h+⁢(Es-i⁢Γs2)

by analytic continuation. It is straightforward to extend partitioning technique to a reference space of arbitrary high dimensions of square integrable basis functions. The Feshbach-Fano method aims at solving the resonance problem by defining the effective operator *H*_eff_ = *OHO* + *OHT*(*z*)*HO*, where *O*projects onto a given set of square integrable functions. Despite its main capabilities there are two major drawbacks as regards the definition of complex resonances on the so-called unphysical Riemann sheet of the complex energy plane. They are (i) the resonance should be independent of the choices of *O* and *P*, (ii) the real and the imaginary parts must be continued analytically to satisfy Eq. (13). The Balslev-Combes theorem guarantees a more general spectral classification including the existence of resonances corresponding to the analytic continuations, Eq. (13).

Next, we will focus on the actual operators, their domains and ranges. Note that the scaling operation


U(θ)=eiAθ;A=12∑k=1N[ p→kx→k+x→kp→k]

where ${\vec{x}}_{k},{\vec{p}}_{k}$ are the *N* coordinates and momenta of the particles constituting the system, and A the generator of the transformation, is unitary if θ is a real parameter. However, for θ⟶*i*θ, i.e., producing a complex scaling, *U*(*i*θ) will be unbounded. Hence its domain and range need to be carefully defined to accommodate the kinetic and potential ingredients of the Hamiltonian-Liouvillian. Restricting the Hilbert space to an appropriate dense subset (Nelson, 1959), the operations can be carried out for the whole (dilatation analytic) family of operators essentially employed in quantum molecular chemistry, which after extension to the whole Hilbert space yields a transformed Schrödinger equation, displaying its characteristic rotational properties (see Figure 3). For more details regarding its use in quantum chemical applications (see Brändas, 1997, 2012).

One might enquire whether such complex resonance eigenvalues and eigenvectors really exists and furthermore how to ensure their location and representation. First it is possible to formulate exact mathematical conditions that, via a simple projection operator approach, by encircling a specific area of the complex energy plane by a nonintersecting connected test curve, will guarantee spectral contributions of the operator, here the rotated Hamiltonian (Engdahl and Brändas, 1988). Furthermore higher order poles of the resolvent are intrinsically revealed by the structural properties of the bracketing function *h*(*z*). For instance if the resonance condition


$$z_{0}=h\,\left(z_{0}\right)$$

is accompanied by the relation


$$1-h^{\prime }\,\left(z_{0}\right)=0$$

where the residue of (*z*−*h*(*z*))^−1^ at *z* = *z*_0_ equals ⟨Ψ(*z*_0_)|Ψ(*z*_0_)⟩ = 1−*h*′(*z*_0_), one finds that a higher order pole of order *n* = 2 (or higher) exists leading to a linear term in *t* of ψ(*t*) (Brändas, 1976). Additional conditions might be derived to verify the order of the resonance eigenvalue. Since the complex scaled Hamiltonian no longer commutes with its adjoint, i.e., is non-normal, its time evolution under certain conditions might be contractive, for details see Kumicak and Brändas (1993).

Considering finally the relationship between the Schrödinger equation and the quantum-classical Liouville equation (Prigogine, 1980, 1996), distinct representations in terms of irreducible Jordan blocks emanate. Adapting dilatation techniques to the quantum Liouville equation (Obcemea and Brändas, 1983), one finds a qualitatively different pole behavior (Figure 2). In particular one is able to identify steady state boundary conditions for the generation of entropy production and associated negentropic gain. The boundary condition at *dS* = 0, where *S* is the entropy of the open system, provides the transition density matrix, ρ_tr_, presented in the introduction, with its nilpotent character, dimension *n*, tuned to the actual temperature and the associated time scales of the process (Brändas, 2019, 2021). These types of constraints are often engaged in a trade-off between entropy production and loss, and explored as an informatic theoretic approach (Bernstein and Levine, 1972). The concept of surprisal has recently been developed and utilized in neuroscience (Friston, 2010; Solms, 2018), see also additional comments and observations (Brändas and Poznanski, 2020).

One of the most intricate consequences of the negentropic entanglement is the transformation ***B*** that organizes the thermally excited density matrix to its classical canonical form, ω = *e*^*i*π/*n*^,


$$\text{B}=\frac{1}{\sqrt{n}}\,\left(\begin{array}{ccccc} 1 & \omega & \omega^{2} & \cdot & \omega^{n-1}\\ 1 & \omega^{3} & \omega^{6} & \cdot & \omega^{3\,\left(n-1\right)}\\ \cdot & \cdot & \cdot & \cdot & \cdot \\ \cdot & \cdot & \cdot & \cdot & \cdot \\ 1 & \omega^{2\,n-1} & \omega^{2\,\left(2\,n-1\right)} & \cdot & \omega^{\left(n-1\right)\,\left(2\,n-1\right)} \end{array}\right)$$

Restated, it brings the density matrix, represented as a complex symmetric matrix (Reid and Brändas, 1989), quantum-thermally excited and constrained to the appropriate temperature with the Segrè characteristic equal to *n*, to the nilpotent matrix **J**, discussed in the introduction. This miracle depends on three crucial observations: (1) the open system dynamics compels a non-Hermitian extension (Balslev and Combes, 1971), (2) quantum correlations and thermal fluctuations interfuse to create a correlated dissipative structure with the capacity to self-organize (Chatzidimitriou-Dreismann and Brändas, 1991), and finally (3) the resulting complex symmetric form is brought to canonical form by ***B***, see the discussion in the previous section (Reid and Brändas, 1989). The factoring properties of the columns of ***B*** suggest a syntax, i.e., the thermo-qubit, for communication between life forms, denoted Off-Diagonal Long-Range Correlated Information, ODLCI in Poznanski and Brändas (2020), as an extension of Yang’s Off-Diagonal Long-Range Order ODLRO (Yang, 1962). The prime number coding, reminding on Gödel numbering, represents a primary thermo-qubit of fundamental physically objective interactions-correlations, which, via entropy lowering, balancing entropy production, extends to the genetic cellular machinery and, what is more, to subjective semiotic communications with semantic content, and ultimately to consciousness and human intelligence.

## Conclusion

We have not mentioned Erwin Schrödinger’s early efforts in 1944, comparing life with its quantum molecular information stored in an aperiodic crystal, see the Canto edition (Schrödinger, 1992). The historic development from Lamarck, via Darwin and Schrödinger to Monod (1971), has recently been reviewed by Maruani (2020, 2021), while attempting to find a biological Lagrangian operator to define a suitable fitness functional to reach a consistent evolution functional, performing deconvolution using Fourier Transforms. Moving beyond conventional quantum waves, the pitch waves built from low-frequency quasi-musical waves, being transcriptions of nucleic acid or protein patterns, are assigned a higher level informational quality compared to the thermally related oscillations. The music of the genes might perhaps in some way correlate with the steady state negentropic coherence of the correlated dissipative structures discussed above.

As pointed out, the derivation of these coherent structures and their properties has not been at the center of attention here. We refer to the personal reference list below for more details. Instead our focus has been concentrated on the particularities of the Fourier-Laplace transform. Notably, the transform relates conjugate observables, such as energy-time, momentum-space, phase and particle number, and temperature-entropy. The adaptation to the underlying structure of linear algebra, in concert with rigorous extensions to incorporate non-normal operators and their generalized spectral properties, add structural regularity and novel irreducible symmetries to the formulation. The Fourier-Laplace resolvent-propagator relationship simplifies to mnemotechnic algebraic reductions mirroring their conjoined spectral representations commensurate with their original conjugate connection as detailed earlier earlier in the section “The Fourier-Laplace Transform.”

The presentation reveals a deeper protocol, viz. physical reality and the role of the observer in axiomatic quantum theory. Löwdin (1992, 1998) investigated the mathematical tools in natural sciences particularly in connection with the properties of linear spaces. A key quantity is here the abstract metric of the linear space and its binary product. For instance the brain’s capability to isolate similarities and differences has been claimed to be essentially equivalent to the brain performing a Gram-Schmidt orthogonalization (Srivastava and Sampath, 2017). A general quantum theoretical formulation must accordingly be built on a solid axiomatic operator algebra in analogy with von Neumann’s philosophy of dealing with an ensemble of physical systems. The essay Löwdin (1992), dedicated to Sir Karl Popper on account of his ninetieth birthday, concludes with an appraisal that the whole of quantum chemistry might consistently be built from four simple axioms subject to a positive definite binary product, but with an intriguing twist. Even if physical interpretations cannot have a direct physical reality belonging to a more or less “contentless” mathematical structure, there is the Gödelian branching point of abstract theories, the genetic dogma and the riddle of life. Although not explicitly spelled out in the thesis, the possibility of a non-positive definite scalar product and an extension to include Einstein’s theory of relativity is ambient and captivating.

An answer to unblock the confounding factors of the mind’s digital fortress has been attempted in this contribution, i.e., by incorporating the self-referential paradox through a dilation analytic binary product in concert with the metric properties maintained and preserved in the theory of special and general relativity (Brändas, 2016). The inherent difficulty of uncovering irreducible degeneracies, i.e., Jordan blocks, proves to be a blessing in disguise affording higher order analytic structures in the evolution of biological systems. The need for a consistent evaluation of the conjugate operator representations has been investigated and analyzed in some detail. It is quite surprising to realize the consequences offered by the change from a positive to a non-positive definite metric. Not only becomes relativity, self-references and in general telicity, the latter referring to processes owing their goal-directedness to the influence of an evolved program (Mayr, 2004), conceivable, but the formulation unfolds a syntax that organizes communication simpliciter, i.e., communication restricted semiotically to exclude semantics. The pragmatic use of semantic communication adds model dependent axioms to the table in agreement with the Gödelian bifurcation of any conceptual model. The description entails an extension to open system dynamics providing a self-referential amplification underpinning the signature of life as well as the evolution of consciousness via long-range correlative information, ODLCI. More recently this relation has been presented as a mind-brain doctrine denoted Panexperiential Materialism (Poznanski and Brändas, 2020).

## Data Availability Statement

The original contributions presented in the study are included in the article/supplementary material, further inquiries can be directed to the corresponding author/s.

## Author Contributions

The author confirms being the sole contributor of this work and has approved it for publication.

## Conflict of Interest

The author declares that the research was conducted in the absence of any commercial or financial relationships that could be construed as a potential conflict of interest.

## Publisher’s Note

All claims expressed in this article are solely those of the authors and do not necessarily represent those of their affiliated organizations, or those of the publisher, the editors and the reviewers. Any product that may be evaluated in this article, or claim that may be made by its manufacturer, is not guaranteed or endorsed by the publisher.
